# *FGFR2*-amplified tumor clones are markedly heterogeneously distributed in carcinomas of the upper gastrointestinal tract

**DOI:** 10.1007/s00432-022-04460-w

**Published:** 2022-11-23

**Authors:** Jan Albin, Luca Fahrig, Janna Siemanowski, Jan Rehkaemper, Florian Gebauer, Thomas Zander, Reinhard Buettner, Christiane Josephine Bruns, Wolfgang Schroeder, Hakan Alakus, Lena Hieggelke, Alexander Quaas

**Affiliations:** 1 Department of General, Visceral and Cancer Surgery, University Hospital Cologne, Cologne, Germany; 2 Institute of Pathology, University Hospital Cologne, Kerpener Street 62, 50937 Cologne, Germany; 3 Department of Internal Medicine I, University Hospital Cologne, Cologne, Germany

**Keywords:** FGFR2 amplification, Tumor heterogeneity, Esophageal adenocarcinoma, Gastric carcinoma, Morphological heterogeneity

## Abstract

**Background:**

FGFR2 is a therapy-relevant target in tumors of the upper gastrointestinal tract (GIT), and clinical trials are currently underway to test the efficacy of FGFR2 inhibitors. Tumor heterogeneity is one of the relevant causes of treatment failure. Almost nothing is known about the heterogeneous distribution of *FGFR2*-amplified clones in adenocarcinomas of the upper GIT.

**Patients and methods:**

To assess *FGFR2* gene copy number alteration and intratumoral heterogeneity of upper GIT adenocarcinomas, we analyzed 893 patient-derived formalin-fixed paraffin-embedded tumor specimens, including primary operated and neoadjuvant-treated tumors (462 gastric carcinomas and 429 esophageal adenocarcinomas) as well as complementary lymph node and distant metastasis by fluorescence in situ hybridization.

**Results:**

Twenty-six gastric tumors (5.6%) and 21 esophageal adenocarcinomas (4.9%) showed *FGFR2* amplification. Overall, 93% of gastric carcinomas and 83% of esophageal carcinomas showed heterogeneous amplification. *FGFR2* amplification was found in different histological growth patterns, including intestinal and diffuse type according to the Lauren classification. In the primary gastric carcinoma group, *FGFR2* amplification was associated with poor prognosis (*p* = 0.005).

**Conclusion:**

Homogeneous *FGFR2* amplification in tumors of the upper GIT is the exception. This has highly relevant implications in the nature of FGFR2 diagnostics (sufficient tumor cell number, determination of amplification at metastasis versus primary tumor, etc.) and on the response probability of appropriate inhibitors. It is relevant that the often poorly treatable and aggressive subtype of diffuse carcinomas (poorly cohesive carcinomas) also shows *FGFR*2 amplification and that an individualized therapy option with FGFR2 inhibitors could be an option in this group.

**Supplementary Information:**

The online version contains supplementary material available at 10.1007/s00432-022-04460-w.

## Introduction

Gastric cancer is the fourth and esophageal cancer is the sixth leading cause of mortality worldwide (Sung et al. 2021). Therapeutic options are still needed to improve the outcome of patients with these aggressive tumors of the upper gastrointestinal tract (GIT); they are often diagnosed at an advanced tumor stage, when curative surgical treatment alone is no longer possible. Fibroblast growth factor receptor 2 (FGFR2) is emerging as a promising target for personalized therapies alongside other targets such as human epidermal growth factor receptor 2 (HER2)/neu, epidermal growth factor receptor (EGFR), and vascular endothelial growth factor receptor (VEGFR). FGFR2 belongs to the family of fibroblast growth factor receptor tyrosine kinases (RTKs), which activate various downstream pathways after binding their ligands.(Lau et al. 2018) Abnormal FGFR signaling pathway activation is known to drive cancer development through increased cell proliferation, prolonged survival, and angiogenesis, and it promotes metastasis (Turner and Grose 2010).

*FGFR2* amplification is the most common genetic alteration of FGFR genes in gastric cancer (Cristescu et al. 2015; Gu et al. 2021). It has also been detected in a share of gastroesophageal adenocarcinomas (Klempner et al. 2019). According to the results of larger case series, the proportion of *FGFR2-*amplified tumors of these entities is around 4–7% (Klempner et al. 2019; Hur et al. 2020; Matsumoto et al. 2012; O’Sullivan et al. 2014) Two splice variants of *FGFR2* have been described: FGFR2b and FGFR2c (Miki et al. 1992).

*FGFR2* amplification leads to specific overexpression of the FGFR2b isoform of the receptor in GIT carcinomas (Gemo et al. 2014; Pierce et al. 2014; Ahn et al. 2016). FGFR2c overexpression has only been described in a small proportion (0.7%) of *FGFR2*-amplified and FGFR2b-overexpressing GIT carcinomas, and thus its role as an independent prognostic factor is questionable (Yashiro et al. 2021). Bemarituzumab, an afucosylated monoclonal antibody against FGFR2b, has shown promising results in clinical trials as a targeted therapeutic for patients with *FGFR2*-amplified and/or FGFR2b-overexpressing and HER2-negative metastatic and locally advanced upper gastrointestinal adenocarcinoma (Catenacci et al. 2019; Wainberg et al. 2021).

Biomarkers with high sensitivity and specificity as well as a precise knowledge of the tumor composition are essential for the success of such specific therapy. Intratumoral heterogeneity is frequently observed in adenocarcinomas of the upper GIT (Grillo et al. 2016; Zubarayev et al. 2019). Thus, the failure of the Gatsby study is most likely due to the intratumoral heterogeneity of Her2/neu in gastric cancer (Thuss-Patience et al. 2017). The intratumoral heterogeneity of *FGFR2*-amplified adenocarcinomas of the upper GIT has been investigated in only a few studies using different methods (Ye et al. 2015; Pectasides et al. 2018; Schrumpf et al. 2022; Tokunaga et al. 2016; Kuboki et al. 2018). Fluorescence in situ hybridization (FISH) is considered the gold standard to detect copy-number alterations (CNA) in tumor cells despite technical alternatives. (F)ISH is the only technique that can reliably detect potential CNA heterogeneity within the tumor.

The previously published results on the distribution of FGFR2b overexpression/*FGFR2* amplification in the different histological types according to the Lauren classification have been inconsistent. Recently, researchers have suggested an association between FGFR2b overexpression/*FGFR2* amplification and intestinal phenotype and lower tumor grade (Schrumpf et al. 2022), while others have described a significant correlation with the diffuse histological type (Matsumoto et al. 2012; Ahn et al. 2016) or even no association with the histological subtype (O'Sullivan et al. 2014; Shoji et al. 2015).

The present study addresses the following questions: (a) How frequently is heterogeneous *FGFR2* amplification found in adenocarcinomas of the upper GIT? (b) What are the morphological characteristics of *FGFR2*-amplified carcinomas? (c) What is the actual relevance of *FGFR2* amplification in adenocarcinoma of the esophagus? (d) What is the impact of neoadjuvant therapy on *FGFR2* amplification? To answer these questions, we used FISH to investigate 462 gastric carcinomas and adenocarcinomas of the gastroesophageal junction and 429 adenocarcinomas of the esophagus as well as complementary lymph node metastases of Caucasian patients.

## Patients and methods

### The gastric carcinoma cohort

The gastric carcinoma cohort consisted of 462 patients. Of these, 272 (58.9%) underwent primary surgery and 190 (41.1%) received neoadjuvant therapy before surgery (Table 1).

**Table 1:** Patient and tumor characteristics of gastric carcinomas

	Overall collective (*n* = 462)		Non-amplified (*n* = 436)		Amplified (*n* = 26)	
Sex						
Male	314	68.0%	296	67.9%	18	69.2%
Female	148	32.0%	140	32.1%	8	30.8%
Preoperative treatment						
None	272	58.9%	259	59.4%	13	50.0%
Neoadjuvant	190	41.1%	177	40.6%	13	50.0%
Age						
< 45	39	8.4%	36	8.3%	3	11.5%
> 45	423	91.6%	400	91.7%	23	88.5%
UICC Stage						
(y)1	94	20.3%	93	21.3%	1	3.8%
(y)2	127	27.5%	119	27.3%	8	30.8%
(y)3	162	35.1%	151	34.6%	11	42.3%
(y)4	79	17.1%	73	16.8%	6	23.1%
Molecular subtype						
CIN	354	76.6%	332	76.1%	22	84.6%
GS	50	10.8%	48	10.9%	2	7.7%
MSI	36	7.8%	34	7.9%	2	7.7%
EBV	22	4.8%	22	5.1%	0	0.0%
Lauren classification						
Intestinal	187	40.5%	174	39.9%	13	50.0%
Diffuse	225	48.7%	212	48.6%	13	50.0%
Mixed	50	10.8%	50	11.5%	0	0.0%
Localisation						
GEJ	151	32.7%	143	32.7%	8	30.8%
Proximal	43	9.3%	40	9.2%	3	11.5%
Corpus	123	26.6%	117	26.8%	6	23.1%
Distal	103	22.3%	95	21.9%	8	30.8%
Other	42	9.1%	41	9.4%	1	3.8%

462 patients were analyzed

Standardized surgical treatment included subtotal distal or total gastrectomy with trans-hiatal resection of the distal esophagus in the case of an adenocarcinoma of the gastroesophageal junction (Siewert 2), and a systematic D2 lymphadenectomy with the goal of complete resection (R0). Roux-en-Y jejunal loop with gastrojejunostomy was considered the method of choice in the reconstruction procedures.

In the subgroup that received neoadjuvant treatment, three different regimens were used (PFL, MAGIC, FLOT, considering that the patients have been treated over the last 20 years). The majority of the patients were treated according to the MAGIC and FLOT protocols.

Molecular subtyping of gastric carcinomas into the four The Cancer Genome Atlas (TCGA)-defined subtypes (CIN, GS, MSI, and EBV) was performed as described previously (Quaas et al. 2021).

### The esophageal adenocarcinoma cohort

This cohort consisted of 429 patients, also divided into (a) patients receiving primary resection (*n* = 177, 41.4%) and (b) patients receiving neoadjuvant treatment (*n* = 252, 58.6%) (Table 2).

**Table 2:** Patient and tumor characteristics of esophageal adenocarcinomas

	Overall collective (*n* = 429)		Non-amplified (*n* = 408)		Amplified (*n* = 21)	
Sex						
Male	385	90.1%	365	89.5%	20	97.3%
Female	44	10.3%	43	10.5%	1	4.9%
Preoperative treatment						
None	177	41.4%	164	40.2%	13	61.9%
Neoadjuvant	252	58.6%	244	59.8%	8	38.1%
Age						
< 65	220	51.4%	212	51.9%	8	38.1%
> 65	208	48.5%	196	48.0%	13	61.9%
Tumor Stage						
(y)pT1	92	21.5%	90	22.1%	2	9.5%
(y)pT2	82	19.2%	77	18.9%	5	23.8%
(y)pT3	245	57.0%	231	56.6%	13	61.9%
(y)pT4	11	2.6%	10	2.5%	1	4.8%
Lymph node metastasis						
(y)pN0	170	39.6%	164	40.2%	6	28.6%
(y)pN1	159	37.1%	151	37.0%	8	38.1%
(y)pN2	53	12.4%	53	13.0%	0	0.0%
(y)pN3	47	11.0%	40	9.8%	7	33.3%

429 patients were analyzed

The standard surgical procedure was laparoscopic gastrolysis and right transthoracic *en bloc* esophagectomy including two-field lymphadenectomy of mediastinal and abdominal lymph nodes. Reconstruction was performed by high intrathoracic esophagogastrostomy as described previously (Holscher et al. 2007). Patients with advanced esophageal cancer (cT3, cNx, M0) received preoperative chemoradiation (5-fluorouracil, cisplatin, and 40 Gy, provided to patient treated prior to the CROSS trial) or chemotherapy alone.

During the first 2 years, patients were followed up clinically in the hospital every 3 months. Subsequently, annual exams were carried out. Follow-up examinations included a detailed history, clinical evaluation, abdominal ultrasound, chest X-ray, and additional diagnostic procedures as required. Follow-up data were available for all patients. There was a preponderance of minor responders based on the tissue microarrays (TMAs), defined as histopathological residual tumor of ≥ 10% (Schneider et al. 2008).

### TMA construction

For TMAs, 1 tissue core (and up to 12 tissue cores for multi-spot TMAs; see below) from each tumor was punched out and transferred to a TMA recipient block. Each TMA was constructed as described previously (Simon et al. 2004; Helbig et al. 2016). In brief, one tissue cylinder with a diameter of 1.2 mm was punched from selected tumor tissue blocks using a self-constructed, semi-automated precision instrument and embedded in empty recipient paraffin blocks.

Consecutive sections of the resulting TMA blocks were transferred to an adhesive-coated slide system (Instrumedics Inc., Hackensack, NJ, USA) for immunohistochemistry and FISH. See the following subsection for details on how we constructed the heterogeneity array (multi-spot TMA).

### Heterogeneity of FGFR2 amplification

To address the question of heterogeneous *FGFR2* amplification within a tumor, we studied primary resected tumors. There were 119 evaluable primary operated gastric carcinomas and 144 adenocarcinomas of the esophagus with corresponding lymph node metastases available for single-spot TMAs. This investigation could answer the following question: How often do different amplification results occur between the primary tumor and its lymph node metastases?

Out of the above-mentioned samples, we analyzed 21 different tumors using whole tumor blocks (10 paired as well as 5 unpaired primary gastric tumors and 5 paired as well as 1 unpaired primary esophageal tumors).

In an additional step, we used a multi-spot TMA considering primary resected esophageal adenocarcinomas as described previously (Gebauer et al. 2020). Briefly, we punched 12 tumor spots out of the same tumor, 4 spots each from the surface and the invasion front and 4 spots from corresponding lymph node metastases. We examined 29 tumors with multi-spot TMA. Twenty-eight of these tumors had previously been analyzed with single-spot TMA and were now being evaluate for possible heterogeneous *FGFR2* amplification in the tumor using multi-spot TMA. We did not view any of the tumors analyzed with multi-spot TMA on whole tumor blocks.

**Fig. 1:**
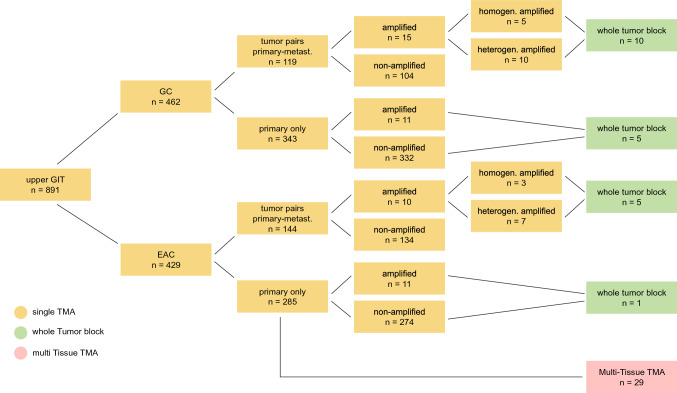
Structure of the analyzed tumor collective: A total of 891 adenocarcinomas of the upper gastrointestinal tract were analyzed by fluorescence in-situ hybridization (FISH) (462 gastric carcinomas, 429 adenocarcinomas of the esophagus). For this purpose, a tissue micro array (single-spot TMA = highlighted in yellow) was used, which considered a tumor spot from the primary tumor and an additional tumor spot from metastases where present (gastric carcinomas: 343 primary tumors without corresponding metastases and 119 tumors in which both the primary tumor and a corresponding metastasis were present. Esophageal carcinomas: 285 primary tumors without corresponding metastases and 144 tumors in which both the primary tumor and a corresponding metastasis were present). Of 462 gastric carcinomas analyzed, 26 tumors showed *FGFR2* amplification (5.6%). Of 429 esophageal adenocarcinomas analyzed, 21 tumors showed *FGFR2* amplification (4.9%). To better reflect tumor heterogeneity, we performed supplemental analysis of 50 tumors. 15 tumors of the stomach were analyzed on large tumor areas (= marked in green) (of which 10 were primary tumors and their corresponding metastases), 6 tumors of the esophagus were analyzed on large tumor areas (= marked in green), and 29 esophageal carcinomas were analyzed with a multi-spot TMA (= marked in red), which included 12 tumor spots from different tumor areas and corresponding metastases. 49 of these supplementary analyzed tumors were already represented on the single-spot TMA, and one additional esophageal carcinoma was considered on the multi-spot TMA

**Fig. 2:**
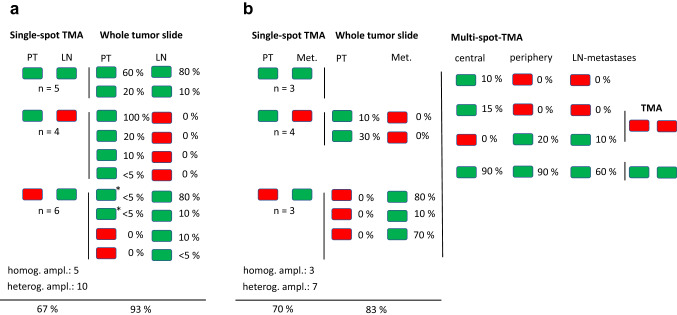
Heterogeneity of *FGFR2* amplification: **a** Heterogeneity of *FGFR2* amplification in gastric cancer (green amplified, red not amplified). Left Heterogeneity between primary tumor (PT) and lymph node/distant metastasis was analyzed on tissue micro array (TMA). Of 119 tumor/metastasis pairs 15 showed FGFR2 amplification, 67% of these were heterogenous amplified. Right: Whole tumor slides of ten tumor/metastasis pairs were analyzed for percentage of FGFR2 amplified tumor cells. Two primary tumor samples without amplification on TMA showed FGFR2 amplified tumor clones on the whole slide (*). One primary tumor showed homogenous amplification in all tumor cells (= 100%). **b** Heterogeneity of *FGFR2* amplification in esophageal adenocarcinoma (green = amplified, red = not amplified). Left Heterogeneity between primary tumor (PT) and lymph node/distant metastasis (Met) was analyzed on single-spot tissue micro array (single-TMA). Of 144 tumor/metastasis pairs ten showed *FGFR2* amplification, 70% of these were heterogenous amplified. Middle Whole tumor slides of five tumor/metastasis pairs were analyzed for percentage of *FGFR2* amplified tumor cells. Only one primary tumor showed homogenous amplification in all tumor cells. Right 4/29 tumors at the multi-spot TMA showed *FGFR2* amplification. Only one tumor was homogeneously amplified in different parts of the primary tumor as in the metastasis and also showed a high proportion of amplified tumor clones within the tumor

In summary, we analyzed 891 carcinomas. In 263 tumors, both the primary tumor and corresponding metastases were present, and we also analyzed 50 tumors with whole tumor blocks or multi-spot TMA (Figs. 1 and 2).

### FISH

We used FISH to evaluate the *FGFR2* amplification status using the Zytolight SPEC FGFR2/CEN 10 Dual Color Probe (Zytovision, Germany) according to the manufacturer’s protocol. We processed samples as described previously (Loeser et al. 2017). We scanned tumor tissue for gene copy gains including chromosomal cluster amplifications hot spots using a 63 × objective (DM5500 fluorescent microscope; Leica, Germany). If the signals were distributed homogeneously, then we used random areas to count the signals. We evaluated 20 tumor cells by counting green *FGFR2* and orange centromere signals. The reading strategy for detecting amplifications followed the recommendations of an *FGFR2*/*CEN10* ratio > 2.0 or *FGFR2* extrachromosomal cluster amplification signals (O’Sullivan et al. 2014). Because there are still insufficient data on the distribution of *FGFR2*-amplified tumor cells in GIT adenocarcinomas, we semi-quantitatively documented all amplified tumor cells as a percentage of all tumor cells examined in a sample.

### Statistical analysis

We collected patient data prospectively. We compared interdependence between staining, tumor characteristics, and clinical data using Pearson’s chi-squared test and Fisher’s exact test, illustrated by cross-tables. We evaluated overall survival from the date of surgery until death. We generated Kaplan–Meier curves and compared them using a log-rank test. We censored patient data with no events or loss to follow-up at the last known date. We performed multivariate analysis for prognostic factors using a Cox regression model. We included factors that could potentially affect survival. Specifically, we used ENTER because this method inserts all variables into the model at the same time. We considered a two-sided *p* < 0.05 to be statistically significant. We used SPSS Statistics Version 25 (IBM, Armonk, NY, USA) for all statistical analyses.

## Results

### Gastric carcinoma

Patient and tumor characteristics of the overall gastric carcinoma cohort.

The majority of patients were male (68%) and over 45 years of age (91.6%). Advanced International Union against Cancer (UICC) stages 3 and 4 were documented in 52.2% of cases. Proximal carcinomas were more frequent (42%) than distal carcinomas (22.3%). According to the four molecular subtypes defined by TCGA, the majority of tumors belonged to the CIN subgroup (76.1%), followed by GS tumors (10.8%), MSI tumors (7.8%), and EBV-positive tumors (4.8%). Overall, 41.1% of patients received neoadjuvant therapy.

### Patient and tumor characteristics of the *FGFR2*-amplified gastric carcinoma

This cohort included 26 amplified tumors (5.6% of the total gastric carcinoma cohort). The sex ratio of *FGFR2*-amplified gastric tumors was comparable to the overall cohort. There was an equal number of tumors with (13/26) or without (13/26) neoadjuvant therapy. There was an equal proportion of tumors with diffuse (13/26) and intestinal (13/26) histology according to the Lauren classification. However, the proportion of *FGFR2*-amplified tumors in the overall cohort was slightly higher in the group of gastric carcinomas with intestinal histology (13/187, 7.0%) than that of tumors with diffuse morphology (13/225, 5.1%), possibly due to the distribution of the investigated subtypes. *FGFR2*-amplified tumors were more distally localized (30.8%). According to the general dominance of CIN tumors, most *FGFR2*-amplified tumors (22/26, 84.6%) could be assigned to this subtype. There was co-amplification of *ERBB2*/Her2/neu in 9.4% of *FGFR2*-amplified tumors. In the neoadjuvant treated, *FGFR2*-amplified group (13/26), as expected, young patients (< 45 years) were slightly more frequent (Table 1). Otherwise, there were no relevant differences between previously treated and treatment-naïve patients.

**Fig. 3:**
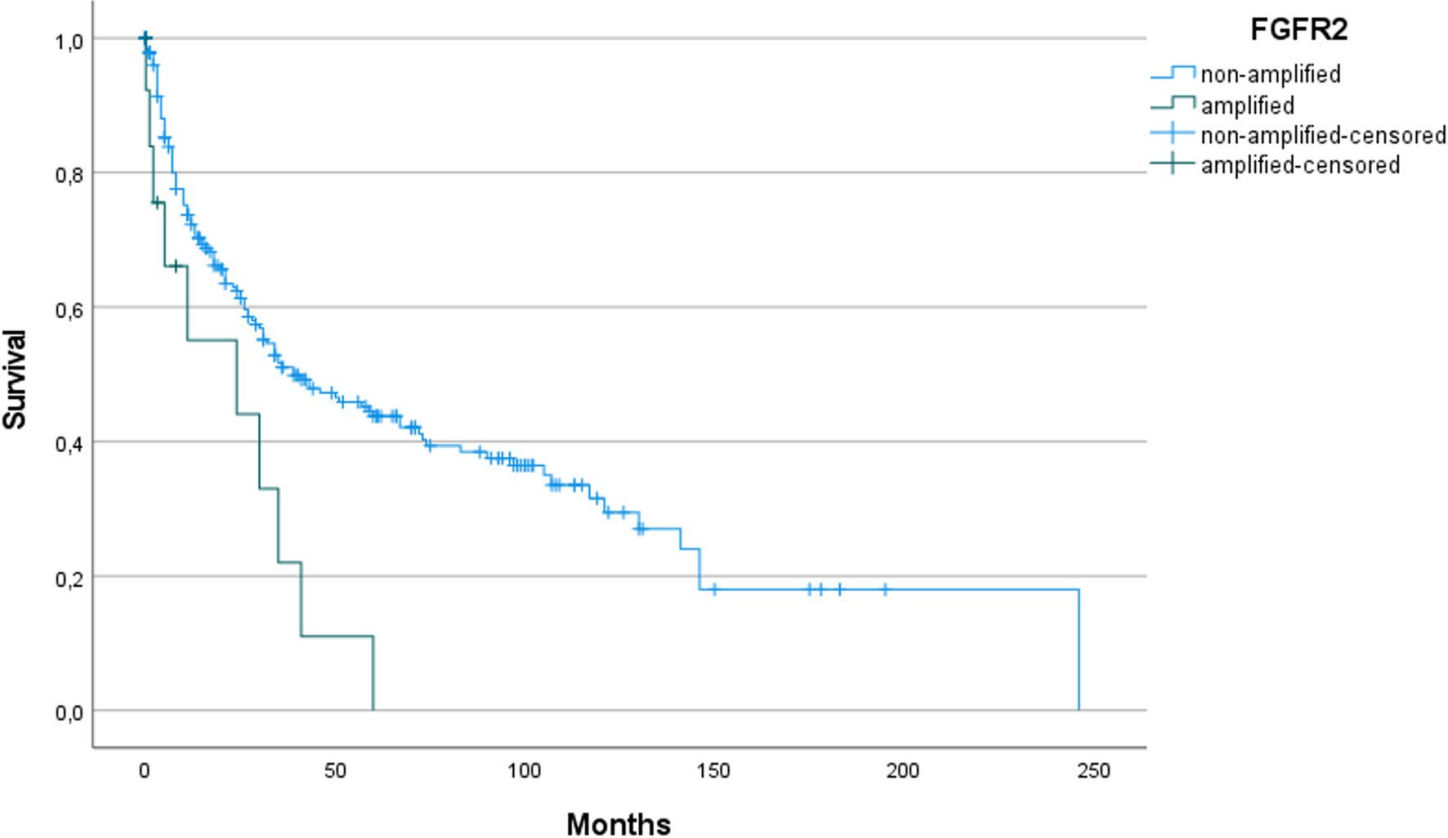
Kaplan–Meier curve: in the primary gastric cancer group, patients with *FGFR2* amplified tumors show significantly worse survival (*p* = 0.005)

*FGFR2* amplification had a prognostically unfavorable effect (*p* = 0.005) in the group of primary operated gastric carcinomas (Fig. 3). This negative prognostic effect was no longer measurable in the neoadjuvant-treated group.

### Esophageal adenocarcinoma

#### Patient characteristics of the overall esophageal adenocarcinoma cohort

The cohort contained predominantly male patients (385/429, 90.1%). Advanced tumor stages—(y)UICC stages 3 and 4—represented more than half of the cases (66.7%, Table 2). Primary resected and neoadjuvant-treated patients showed similar distributions regarding sex, age, and UICC stage (lower proportion of UICC stages 3–4 in the primary resected subgroup).

#### Patient characteristics of the *FGFR2*-amplified esophageal adenocarcinoma cohort

**Fig. 4:**
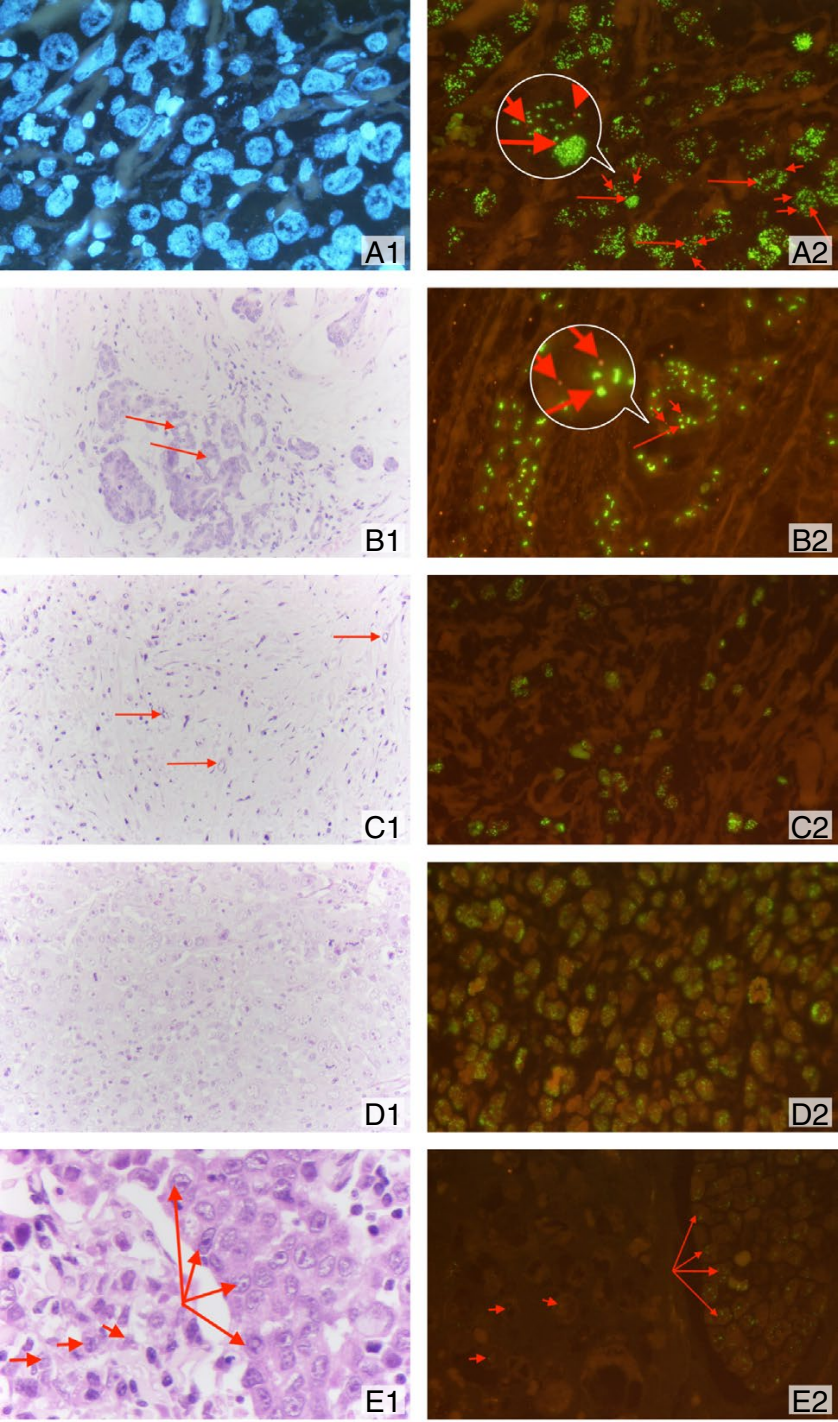
Different tumor growth patterns with *FGFR2* amplification and intra-tumoral heterogeneity of *FGFR2* amplification. In A1: Adenocarcinoma with DAPI signal (FISH), A2 shows cluster amplification (green signals, long arrow) and two red signals of chromosome centromeres (short arrow). In B1, hematoxylin–eosin standard (H&E) staining of a tubular and partially mucinous adenocarcinoma *FGFR2-*amplified (B2, cluster amplification with green signals (long arrow) and centromere signal in red (short arrow). In C1 and C2, poorly cohesive adenocarcinoma with *FGFR2* amplification. In D1 and D2, solid growing carcinoma with medullary features also *FGFR2* amplified. This selection of *FGFR2* amplified carcinomas highlights that morphology cannot be a predictor of this gene-amplification. E1 and E2 exemplify the highly relevant intra-tumoral heterogeneity of FGFR2 amplification. E1 shows on H&E the tumor clones that are *FGFR2* amplified (long arrows), and the *FGFR2* non-amplified tumor portions are shown with short arrows

There were 21 patients in this amplified cohort (4.9% of the total esophageal adenocarcinoma cohort). The sex and age distribution in *FGFR2* amplified tumors was comparable to the overall esophageal adenocarcinoma cohort. As with gastric carcinoma, *FGFR2*-amplified esophageal adenocarcinomas showed different morphological growth patterns, like tubular, mucinous, and poorly cohesive carcinomas (Fig. 4). The proportion of *FGFR2*-amplified adenocarcinomas was higher in treatment-naïve patients (13/144, 7.3%) than in neoadjuvant-treated patients (8/252, 3.2%), resulting in an approximate 1/3 to 2/3 distribution of naïve to neoadjuvant-treated tumors within *FGFR2*-amplified esophageal adenocarcinomas.

Primary operated patients with *FGFR2*-amplified esophageal adenocarcinoma showed a tendency to a worse prognosis (overall survival). However, this did not reach statistical significance (*p* = 0.346), in contrast to patients with gastric carcinoma (Supplemental Figure).

### Heterogeneity of *FGFR2* amplification in upper GIT carcinomas

#### Heterogeneity of the primary tumor and corresponding lymph node metastases on single-spot TMA

From 119 cases with primary gastric carcinoma and corresponding lymph node metastasis, 15 tumor pairs showed *FGFR2* amplification (Figs. 2 and 4). In five cases, both the primary tumor and its lymph node metastasis showed concordant *FGFR2* amplification. Ten tumors showed heterogenous amplification of *FGFR2*: four *FGFR2*-amplified primary tumors lacked *FGFR2* amplification in their lymph node metastases, while six primary tumors lacked *FGFR2* amplification, but the lymph node metastases did have *FGFR2* amplification.

From 144 cases with primary esophageal adenocarcinoma and corresponding lymph node metastases, 10 tumor pairs showed *FGFR2* amplification. Of these, three showed co-amplification in both primary tumors and lymph node metastases and seven showed amplification in either primary tumors or lymph node metastases (four *FGFR2*-amplified primary tumors and three *FGFR2*-amplified lymph node metastases).

Based on the results of single-spot TMA, we analyzed whole tumor blocks of 15 patients with gastric cancer and 6 patients with esophageal cancer as well as 29 additional patients with multi-spot TMA.

#### Heterogeneity of primary tumor and corresponding lymph node metastases on whole tumor blocks and multi-spot TMAs.

Figure 2A summarizes the results of the whole tumor blocks: We analyzed 10 patients with tumor pairs (primary tumor and metastasis) using whole tumor blocks (for a total of 20 tumor slides). Two of the five patients who had *FGFR2* amplification in the primary tumor and metastasis on single-spot TMA were also analyzed with whole tumor blocks and, as expected, showed the same result. However, neither the two primary tumors nor their metastases were homogeneously amplified: they also showed non-amplified tumor cell clones. The percentage indicates the amplified tumor cell content relative to all tumor cells. This may indicate that the amplified tumor cell clones were biologically relevant and induced lymph node metastases. We also evaluated the four patients whose primary tumors showed *FGFR2* amplification on single-spot TMA but did not show amplification in corresponding lymph node metastases by using whole tumor blocks. If the above hypothesis of the biological relevance of *FGFR2*-amplified tumor clones is correct, then lymph node metastases without *FGFR2* amplification could not be easily explained. We confirmed the results from single-spot TMA. Indeed, a tubular adenocarcinoma showed homogeneous *FGFR2* amplification in the primary tumor while the corresponding lymph node metastasis showed no *FGFR2* amplification. We will discuss this aspect in more detail in the Discussion.

Of six patients whose metastases showed *FGFR2*-amplified tumor clones on single-spot TMA but not in the primary tumor, we evaluated four patients with whole tumor blocks. Two primary tumors now had small amplified tumor clusters (< 5% of the total tumor cell number) that were not detected with single-spot TMA and apparently induced the metastases. One gastric carcinoma sample had signs of an early invasive carcinoma and was amplified on TMA. Furthermore, four TMA spots showed few amplified tumor cells. We analyzed these five unpaired gastric carcinoma sample as whole tumor blocks. The early invasive carcinoma and two of four samples with intratumoral heterogeneity on TMA showed *FGFR2* amplification.

#### Esophageal adenocarcinoma

Figure 2B summarizes the results of the whole tumor block analysis of esophageal adenocarcinoma: we considered five patients with corresponding tumor pairs using whole tumor blocks (for a total of ten tumor slides). Two of the four patients whose primary tumors showed *FGFR2* amplification on single-spot TMA but had non-corresponding *FGFR2*-amplified clones in their metastases showed identical results even on whole tumor blocks, and vice versa for the tumors that had only *FGFR2*-amplified clones in the metastases.

We also analyzed one unpaired esophageal carcinoma sample with a whole tumor slide, because it showed only a few amplified tumor cells on single-spot TMA. We confirmed this intratumoral heterogeneity on the larger tissue sample, which showed small clusters of amplified tumor cells.

#### Multi-spot TMAs

We also examined 29 primary operated esophageal adenocarcinomas using a multi-spot TMA that considered eight tumor spots from the primary tumor (4 from the central tumor area and four from the periphery) and 4 spots from corresponding lymph node metastases. None of these tumors were represented on the whole tumor blocks described above. We detected *FGFR2*-amplified tumor cells in 4 of 29 primary tumors. These samples had shown *FGFR2* amplification on single-spot TMA. While *FGFR2*-amplified tumor cells accounted for 10–20% of cells in 3/4 primary tumors and were mainly found in the central tumor area, up to 90% of the infiltrating tumor cells counted were evenly distributed in the center and periphery in 1/4 primary tumors (75% intratumoral heterogeneity). Comparison of 19 primary tumors and their lymph node metastases revealed 100% intrasample heterogeneity (2/2), with a lower proportion of *FGFR2*-amplified tumors in lymph node metastases (90% in primary, 60% in metastases, and 20% primary versus 10% in metastases) (Fig. 2b). We did not find *FGFR2*-amplified tumor cells in lymph node metastases of non-amplified primary tumors.

## Discussion

We analyzed a large number of adenocarcinomas of the stomach (*n* = 462) and esophagus (*n* = 429) from a Caucasian patient cohort (*N* = 891). Our results support pronounced intratumoral heterogeneity of *FGFR2* amplification. Amplification of this gene has been reported in 4–7% of upper GIT carcinomas, with homogeneous *FGFR2* amplification expected in < 20% of these cases (Klempner et al. 2019; Hur et al. 2020; Matsumoto et al. 2012; O'Sullivan et al. 2014). Consistent with these results, we found that 5.3% of patients (47/891) had *FGFR2*-amplified cells. *FGFR2*-amplified gastric carcinomas in our cohort (5.6%) did not show an association with histological subtype or a difference in whether neoadjuvant therapy was used. Our cohort even showed a slightly, although not significantly, higher percentage of *FGFR2*-amplified tumors within the group with diffuse histology compared with the ratio in intestinal tumors. *FGFR2*-amplified esophageal adenocarcinomas (4.9%) showed no specific clinical or histomorphological characteristics. While *FGFR2* amplification in gastric carcinomas had a prognostically unfavorable effect in the primary gastric cancer group (*p* = 0.005), which was not detectable in the neoadjuvant treated group, this effect was not measurable in any subgroup of esophageal adenocarcinomas. This fact may be due to molecular aspects; adenocarcinomas of the esophagus belong molecularly almost exclusively to the group of chromosomally instable carcinomas (CIN), while gastric carcinomas are molecularly more heterogeneous (including a higher proportion of so-called genomically stable tumors (GS)).

To our knowledge, three papers to date has considered tumor heterogeneity of *FGFR2* in gastric cancer (Ye et al. 2015; Pectasides et al. 2018; Schrumpf et al. 2022). Ye et al. (2015) investigated how many endoscopic biopsies are necessary to obtain trustworthy data for biomarkers including HER2/neu and FGFR2. They showed that the probability of false-negative results decreases with the number of biopsies (i.e., the number of tumor cells available for analysis), especially for *FGFR2*. In other studies, the authors showed the same for HER2/neu and programmed death ligand 1 (PD-L1) (Grabsch et al. 2010; Lordick et al. 2017; Schoemig-Markiefka et al. 2021). Pectasides et al. (Pectasides et al. 2018) investigated the heterogeneity of *FGFR2* in the context of other markers such as *ERBB2*, *MET*, and *EGFR*, among others, using other techniques such as next-generation sequencing (NGS) and cell-free DNA (cf-DNA). Consistent with our results, that group also found relevant tumor heterogeneity between primary tumors and metastases, but they also observed a potentially diagnostically important homogeneity between distant metastases and cf-DNA. If our results can be confirmed, liquid biopsies might be able to represent the heterogeneity of *FGFR2* well.

This year, Schrumpf et al. (Schrumpf et al. 2022) published a paper about their analysis of nearly 500 Caucasian gastric carcinomas, studying *FGFR2* mutations and FGFR2 protein expression by immunohistochemistry. In some of the tumors, correlation with protein expression was considered by *FGFR2* chromogenic in situ hybridization (CISH). The authors confirmed high heterogeneity of *FGFR2* expression in gastric carcinomas, a finding that fits excellently with our results. In addition, they found *FGFR2* amplification occurred in poorly cohesive carcinomas.

The heterogeneous distribution of biomarker-positive tumor cell clones in primary tumors and their metastases has both diagnostic and therapeutic important implications. If a genetic alteration, such as *ERBB2*/Her2/neu or *FGFR2* amplification, is homogeneously distributed uniformly across the tumor and its different clones, a single tumor-cell-bearing biopsy would be sufficient to reliably document this genetic alteration. We know Her2/neu amplification can be quite homogenous in come cancer—for example, breast carcinoma (Hanna et al. 2007; Hou et al. 2017). This is not true for Her2/neu amplification in upper GIT adenocarcinomas and, as we have shown in the present study, for *FGFR2* amplification in gastric and esophageal cancer. This finding is relevant in terms of the methods used to determine *FGFR2* amplification. According to Ye et al. (Hur et al. 2020), at least six tumor-bearing biopsies are required to diagnose a negative result (unamplified tumor). Fewer biopsies (e.g. three) increases the risk of a false negative result.

Based on our results, we believe that the primary TMA we used, considering more than 800 samples from carcinoma patients, provided realistic results despite relevant heterogeneity of *FGFR2* amplification. Nevertheless, a single-spot TMA has technical weaknesses in representing tumor heterogeneity. We have responded to these weaknesses with additional methods (whole-tumor block analyses and multi-spot TMA) and believe that we have contributed valid data on *FGFR2* heterogeneity.

Using these measures, we obtained results on our surgical specimens that are in good agreement with the literature. Specifically, 93% of gastric carcinoma and 83% of esophageal adenocarcinoma exhibited intratumoral heterogeneity of *FGFR2* amplification. Furthermore, we demonstrated that there are discrepancies between the amplification status of the primary tumor and the corresponding lymph node metastases, which can be explained by intratumoral heterogeneity of *FGFR2*-amplified tumor clones. This suggests that sufficient sampling in the primary tumor and lymph node or organ metastases is advisable to determine the *FGFR2* status.

For the medical-diagnostic routine, this means that in systemically ill patients who are to receive anti-FGFR2 therapy, FGFR2 biomarker testing should be extended to the biologically driving tumor component, which can also only become apparent in metastases. Alternatively, liquid biopsies could help to reliably determine this systemically relevant status in the future (Pectasides et al. 2018; Wallander et al. 2021). The high intratumoral heterogeneity of *FGFR2* amplification also has implications for the potential efficacy of therapeutic *FGFR2* blockade. Biologically, the more homogeneous a genetic alteration is in a tumor, the more important it is for maintaining tumor integrity. The more homogeneous an oncogenic driver is in a tumor, the more effective its therapeutic blockade is likely to be.

The TOGA study impressively demonstrated the heterogeneous occurrence of HER2/neu-positive tumor cell clones in gastric carcinoma. The high heterogeneity may be one of the reasons why the median survival benefit of Her2/neu blockade with trastuzumab was significant but still moderate at 2.9 months (Cutsem et al. 2015; Ruschoff et al. 2012; Bang et al. 2010). The heterogeneity of Her2/neu may also have been the key reason for the failure of the Gatsby study (Thuss-Patience et al. 2017). The FISH technique we have chosen is particularly good at demonstrating heterogeneous gene amplification, as these different clones are visualized (Fig. 2). Future studies will have to show how well alternative detection methods of an amplification help to map heterogeneity. While the majority of Her2/neu-amplified gastric carcinomas show an intestinal growth pattern (according to the Lauren classification), this is not true for *FGFR2*-amplified tumors. We have shown that half of our *FGFR2*-amplified tumors have a diffuse growth pattern (at least in proportions). Consequently, a clinical trial is currently being initiated to address the efficacy of FGFR2 blockade also in diffuse type carcinomas (Merz et al. 2020).

In the FIGHT study (NCT03343301), a global, randomized, double-blind, placebo-controlled phase 2 trial, patients with unresectable locally advanced or metastatic gastric/-esophageal junction carcinoma were enrolled if their tumor was HER2-negative and positive for FGFR2b overexpression by centrally performed immunohistochemistry or for *FGFR2* amplification determined by circulating tumor DNA (ctDNA) (Catenacci et al. 2019). Generally, it is a method that can be used to detect gene amplification, but the sensitivity is highly dependent on the material used and discordance between other methods has been reported. FISH is the gold standard for the detection of gene amplification. Previously reported FGFR2 immunohistochemistry results did not correlate well with *FGFR2* amplification due to intracellular epitopes and cross-reactivity with other FGFRs, like FGFR1, while a new FGFR2b immunohistochemistry assay has been proposed as an alternative, accurate screening tool (Ahn et al. 2016). Unfortunately, this study antibody is currently not (yet) commercially available. As FISH is the gold standard for amplicon detection and showed the strongest prediction of treatment response in the FIGHT study, we chose this analytical method for our study.

Taken together, *FGFR2*-amplified primary adenocarcinoma of the GIT shows a high rate of heterogeneously distributed tumor clones. This intratumoral heterogeneity is found in both gastric carcinoma and esophageal adenocarcinoma and leads to different expression pattern in metastases. FGFR2 testing in metastases should be performed, because a small aggressive clone of the primary tumor can drive aggressive tumor behavior and tumor spreading. We did not find any significant correlation between a specific histomorphological subtype and *FGFR2* amplification in upper GIT adenocarcinoma; in particular, we found an equal proportion of diffuse and intestinal morphology in *FGFR2*-amplified gastric carcinomas. Therefore, FGFR2 biomarker testing should not be limited to carcinomas of a particular histomorphological subtype. The percentage of *FGFR2*-amplified esophageal adenocarcinoma in our cohort (4.1%) is consistent with the rate reported in the literature for gastric carcinomas. Although there is no significant association with prognosis, testing for *FGFR2* amplification may provide a targeted therapeutic option for these aggressive tumors. According to our data, we could not detect a significant effect of neoadjuvant treatment on *FGFR2*-amplified tumor clones, as we found no significant differences in the distribution of *FGFR2*-amplified tumors in naïve or pretreated adenocarcinomas of the upper GIT.

Sub-analyses of currently ongoing clinical trials investigating the efficacy of FGFR2 or pan-FGFR inhibitors in solid tumors will show the impact of heterogeneity of *FGFR2*-amplified tumor cell clones on treatment response or failure. It is also relevant that the poorly treatable and aggressive subtype of diffuse carcinomas (poorly cohesive carcinomas) shows *FGFR2* amplification. In this group, an individualized therapy with FGFR2 inhibitors might be an option.

## Supplementary Information

Below is the link to the electronic supplementary material.

Supplement Figure Kaplan Meier curve Primarily operated patients with FGFR2 amplified adenocarcinoma of the esophagus show a tendency to a worse prognosis. However, this does not reach statistical significance in contrast to patients with gastric carcinoma (*p* = 0.346) (JPG 36 KB)

## Data Availability

The datasets generated during and/or analysed during the current study are available from the corresponding author on reasonable request.
