# Supplementary figures and images for: FGFR2-amplified tumor clones are markedly heterogeneously distributed in carcinomas of the upper gastrointestinal tract
Source: J Cancer Res Clin Oncol. 2022 Nov 23;149(8):5289–300. doi: 10.1007/s00432-022-04460-w (PMC10349760; doi:10.1007/s00432-022-04460-w)

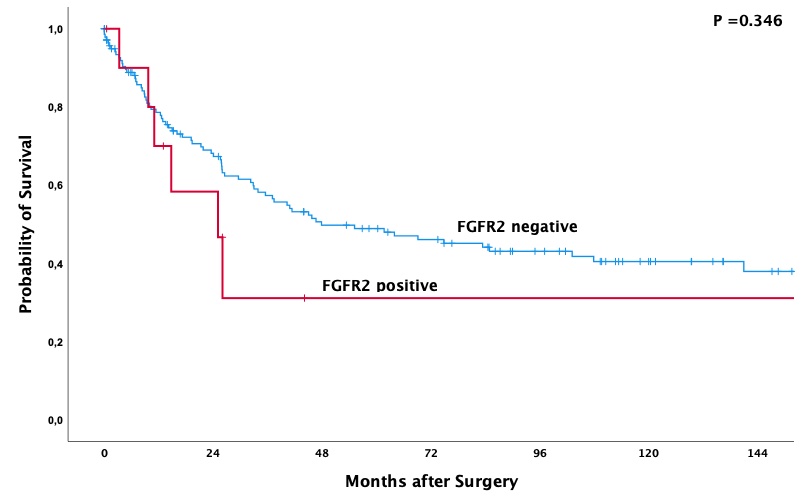

Supplement: Supplementary file 1 — Supplement Figure Kaplan Meier curve Primarily operated patients with FGFR2 amplified adenocarcinoma of the esophagus show a tendency to a worse prognosis. However, this does not reach statistical significance in contrast to patients with gastric carcinoma (p = 0.346) (JPG 36 KB) [file 432_2022_4460_MOESM1_ESM.jpg]
